# Where is an emotion? Using targeted visceroception as a method of improving emotion regulation in healthy participants to inform suicide prevention initiatives: a randomised controlled trial

**DOI:** 10.1186/s13063-020-04479-9

**Published:** 2020-07-14

**Authors:** Steven Davey, Elliot Bell, Jamin Halberstadt, Sunny Collings

**Affiliations:** 1 Suicide and Mental Health Research Group, University of Otago, Wellington, PO Box 7343, Wellington, Newtown 6242 New Zealand; 2 Department of Psychological Medicine, University of Otago, Wellington, New Zealand; 3 Department of Psychology, University of Otago, Dunedin, New Zealand; 4 Suicide and Mental Health Research Group, University of Otago, Wellington, New Zealand

**Keywords:** Emotion regulation, Suicide, Localisation, Interoception, Visceroception

## Abstract

**Background:**

William James’ 1884 paper “What is an emotion?” has generated much recent interest in affective science regarding somatic contributions to emotion. Studies of interoception (“sensing the physiological condition of the body”) suggest that sensing specific parts of the body contributes to the production of emotion, namely when sensing the viscera (i.e. “visceroception” of the heart, gut or lungs). Improved visceroception has, for instance, been linked to increased emotional intensity, suggesting a role for interoception in emotion regulation that may pertain specifically to visceral bodily locations. Thus, in addition to asking James’ question, “*What* is an emotion?”, we ask, “*Where* is an emotion?”. Further, there is an evidence base pointing to the connections between emotion regulation and suicide, and between interoception and suicide. This is a preliminary trial investigating whether targeted interoception/visceroception improves emotion regulation. Ultimately, the overall project aims to inform suicide prevention efforts.

**Methods:**

The trial utilises a pre-test/post-test control group design, with two experimental groups undergoing visceroceptive interventions (focussing on areas pertaining to the gut or heart) and a control group. The interventions will run for 8 weeks. A spatial cueing task will measure reaction times to bodily changes relating to lower abdomen or chest focus. A stop/signal task will measure emotional inhibition, which is hypothesised to obscure awareness of active bodily locations. Visceroceptive ability will be tracked using a heartbeat estimation task, a water load test, and by self-report questionnaire. The sample will consist of healthcare professionals and healthcare students. Despite these being groups that represent a relatively high suicide risk among professional and student groups, all participants will be healthy, given the preliminary nature of this trial.

**Discussion:**

To our knowledge, this will be the first project to address whether emotional feeling presents as a localised bodily phenomenon and whether trained awareness of emotional localisation can improve emotion regulation. It will also be the first to investigate relationships between interoception and emotional inhibition (i.e. whether a sustained interoceptive practice leads to the disinhibition of bodily emotional sensations, which can positively contribute to emotion regulation). These empirical findings on emotion regulation from a healthy sample will be used to inform a desk-based enquiry into the role of embodied emotion in suicide prevention, which may make a significant contribution to a growing evidence base on interoception and suicide.

**Trial registration:**

ACTRN12619000324112. Registered on 4 March 2019. Universal Trial Number (UTN): U1111-1221-0201.

## Background

According to William James in the 1884 paper “What is an emotion?” [1], emotion is defined by, or to a great extent involves, the perception of our body. Some recent developments in affective science take their lead from James [2, 3] in involving a form of body focus named “interoception” (the “sense of the physiological condition of the body” [4]).

There are a number of distinctions to be made to fully define interoception. Firstly, it is distinct from exteroception (i.e. sensing the external environment). Interoception is further differentiated into visceroception (i.e. sensing the viscera) and proprioception (i.e. sensing the skeletal muscles and position of the body). Visceroception is, in turn, to be differentiated into cardioception (i.e. interoception of the heart) and gastroception (i.e. interoception of the gut). There are three dimensions of interoception: sensitivity (i.e. the objective detection of bodily activity), sensibility (i.e. the subjective experience of bodily activity), and metacognitive awareness (i.e. the extent to which sensitivity correlates with sensibility). Much of the emotion research involving interoception has focused on interoceptive—specifically visceroceptive—sensitivity as a measure of interoceptive accuracy, such as in relation to heart beat. Finally, superior visceroception—such as greater accuracy in heart rate detection—has been linked to greater emotional intensity [5], and such improvements in accuracy can be trained [6, 7].

The importance of emotional feeling for well-being has previously been emphasised, with indications that avoidance of negative feeling is linked to psychological disorders [8] and emotion dysregulation [9–11]. Ineffective emotion regulation has in turn been correlated with suicide ideation (i.e. having suicidal thoughts), the desire to attempt suicide [12], and actual suicide attempts [13]. In the specific case of interoception—as one aspect of emotional feeling—low levels have been linked to an increased likelihood of harming oneself [14].

Few studies, however, have addressed this connection between interoception and self-harm or suicide, and those that have were limited to interoceptive sensibility. For instance, Forrest et al. [15] found negative correlations between suicidality and interoceptive sensibility. Forcano et al. [16] reported that women with bulimia nervosa who had attempted suicide had yet greater interoceptive sensibility deficits than those who had not attempted. A more recent study [17] reported that people with lifetime suicide attempts tend to distract themselves from bodily sensations, and those with lifetime suicide ideation reported increased worry about bodily sensations. There was a lack of trust of bodily sensations for participants across the suicidal continuum. Overall, suicidal individuals reported lower levels of interoceptive sensibility than non-suicidal individuals, and this effect was more pronounced at the more extreme (more suicidal) end of the continuum. These authors conclude that “a tendency to ignore or distract oneself from sensations of pain or discomfort may be particularly relevant to the capability for suicide” (p. 7).

To date, no study has investigated whether differential improvements in emotion regulation are possible from practice in the differing forms of visceroception, including cardioception and gastroception. Relatedly, no study has considered whether the embodied aspects that play a part in emotion regulation can be localised in an area of the body corresponding to a form of visceroception, i.e. to cluster in the chest (accessed by cardioception) or the abdomen (accessed by gastroception). This may be especially applicable to the early onset of an emotional response, prior to the development of a “multi-componential syndrome” (see [18]) where body and mind become activated at many locations (e.g. fast heart, clammy palms, increased body temperature, “butterflies” in the stomach), over potentially much longer timescales, and which includes cognitive appraisals of the stimulus. It may be that, during the moments following the presentation of an emotional stimulus or trigger, it is vital to engage in visceroception—as a form of “sensory monitoring” (i.e. the improved “ability to access, accurately identify, regulate, and act on information conveyed in one’s feelings” [19], p. 392) before the emergence of multi-componential syndromes, which may include problematic “emotional cascades” whereby increasingly distressing vicious cycles are generated [20]. If, then, there is a connection between visceroception, improved emotion regulation, and the prevention of cascades, this may be due to improved sensory monitoring of early stage emotional activation in a localised area—a cardioceptive or gastroceptive cluster. Indeed, the gut in particular has been the focus of much emotion research, such as the discovery of the enteric nervous system and gut-brain-emotion interactions [21] including the established gut-emotion connections via the vagus nerve [22].

Finally, there are grounds for thinking that the current lack of evidence regarding localisation may be due to habitual attentional processes. Selective attention involves the concurrent activation of attention to a stimulus and deactivation of attention to a non-stimulus, i.e. attentional inhibition. Decreased interoception may result in inhibited awareness of the physiological condition of the body. This inhibition may ultimately result from the functional dominance of exteroception over interoception during early infancy, i.e. awareness of events in the external world likely has greater survival/social value than the awareness of inner processes; hence, finite attentional resources are allocated accordingly and reinforced over time [23]. Normal development then results in a ubiquitous trade-off between (i) having improved outwardly focused interpersonal/social functioning and (ii) improved personal connection with embodied emotion. With a tendency to orient externally, we may only become aware that our body is active at a later stage of a longer duration multi-componential syndrome and be unable (without training) to attend to an early, brief, principle location of emotional experience. Improvements in interoception over an extended time period may enable identification of location, possibly through a gradual process of disinhibition.

The potential benefit of disinhibition, as opposed to avoidance/inhibition, is the focus of this project, and in particular that a key element may be missing in our current understanding of emotion that is fundamental for emotional feeling: the identification and connection with localised emotion, with a focus on the chest and lower abdomen areas of the body.

### Aims of the project

The overarching aim is to advance understanding of the localisation of emotion via interoceptive training and how this may relate to emotion regulation, with a view to laying the groundwork for the development of clinical interventions pertaining to suicidality.

### Research questions

(RQ1) Is emotional/affective feeling localised?

(RQ2) Can targeted interoception result in indications of localised emotion?

(RQ3) Is the *unlocalisability* of emotional feeling due to inhibition?

(RQ4) What implications does access to localised emotion have for emotion regulation in its relationship with suicide prevention?

## Methods and design

### Design

**Fig. 1:**
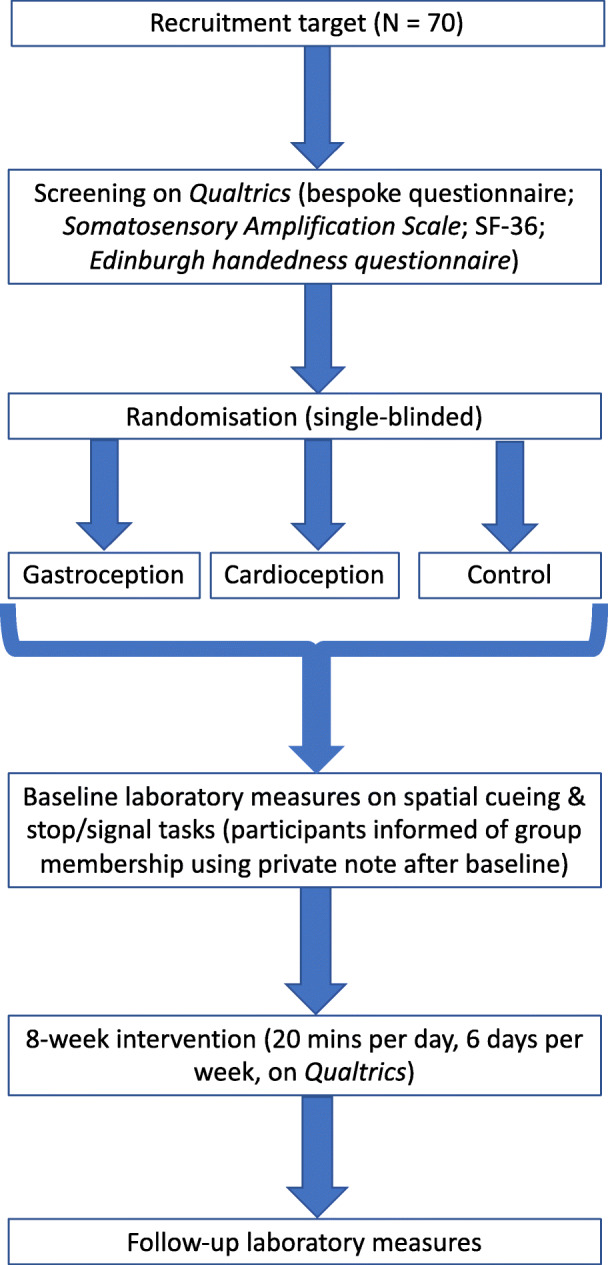
Trial overview

The project will be a parallel group trial using a pre-test/post-test control group design (Fig. 1). There will be two data points (baseline and follow-up) with comparisons between two experimental groups (gastroception vs. cardioception; focussing on the inside of the lower abdomen or chest area, respectively) and a control group.

Since emotion research deals with constructs that are multiply realised [24], the project will employ multiple measures. There will be two primary, single-blinded studies, both laboratory-based, one utilising a spatial cueing task and another utilising a stop/signal task. In addition, physiological and self-report measurements will be taken (these are described in detail below).

### Study population

The sample for the main data collection will be drawn from across the health professions and student healthcare cohorts in the Wellington area, New Zealand. Given the preliminary nature of the trial, only healthy individuals will be recruited at this stage (with health status established by self-report; see below).

Medical students evidently experience difficulties in emotion regulation when dealing with the many stressful situations faced during training [25]. Further, a high prevalence of suicide ideation has been found in medical student populations (e.g. 11% incidence [26]; 15% incidence [27]). Qualified doctors also experience emotion regulation difficulties [28, 29], with male doctors having a suicide rate that is 1.4 times higher than in the general male population, and 2.3 times higher among female doctors than in the general female population [30].

Nurses may also be at greater risk of suicidality, with recent UK statistics [31] indicating this group to be the most at-risk of all health professions [32]. Nursing students may, too, face similar challenges to their medical counterparts.

Finally, allied health professionals—particularly female—have also been identified as at risk [33] and hence will be included in the sample, along with allied health students given that these, too, may have similar experiences to their medical and nursing counterparts.

### Sample size

A power analysis calculation was based on reaction time (RT) measures in Silverstein et al. [34], who reported a mean RT of 2134.1 ms (SD = 856.6 ms) pre-treatment and 1403.6 ms (SD = 462.3 ms) post-treatment for meditators, and 1865.1 ms (SD = 800.4 ms) pre-treatment and 1893 ms (SD = 780.6 ms) post-treatment for controls. This gave a treatment-by-time partial eta-squared of 0.15, with a *p* value of 0.03. Using the same partial eta-squared for the three group, two time point studies, with alpha = 0.05 and 80% power, a minimum of 54 participants would be required in total (across all group conditions). This equates to a minimum of 18 participants per group to detect the main effect in the spatial cueing task. (regarding the stop/signal task, Verbruggen and De Houwer [35] detected effects in a single group with a sample size of 23 participants.) We will aim for 70 participants in total, assuming a drop-out buffer of approximately 5–10 participants. This will allow 20+ participants in each group.

### Recruitment of participants

Potential participants will be recruited by several means. An advertisement (hard copy and electronic form) will be posted around the University of Otago Wellington (UOW), Massey University, Victoria University of Wellington, Wellington Hospital, and the wider District Health Board. In addition, the New Zealand College of Clinical Psychologists and the New Zealand Psychological Society will be approached to attract the interest of clinical psychologists, either in-training or in-post.

The participant information sheet and consent form will be sent to those responding to the advertisement for further consideration. Prior to admission to the research studies, potential participants will also be screened to ensure they meet the inclusion/exclusion criteria.

Screening will be undertaken online (on the survey platform *Qualtrics*) using a brief bespoke questionnaire collecting information on all but two of the inclusion/exclusion criteria (see below) and using the following tools for the remaining criteria:

- *The Somatosensory Amplification Scale* (SSAS) (a brief, 10-item questionnaire: approximately 2 min to complete [36]);
- *The Edinburgh Handedness Questionnaire* [37] (a brief version of this self-report tool will be used: approximately 1 min to complete [38]).

Recruitment of potential participants began in November 2018 and will continue until April/May 2019. Random group allocation will not begin until the middle of May 2019.

### Eligibility criteria

#### Inclusion criteria

Participants must satisfy the following conditions:

- Currently enrolled on a full time medical, nursing or allied healthcare degree/diploma OR already qualified and working as a doctor/nurse/allied healthcare professional;
- Aged 18–35 years. This range is based on indications of declining interoception after approximately 40 years of age [39], and initial declines in RTs have been identified as early as 24 years of age [40]; hence, age is capped at 35 to limit such heterogeneity within the sample;
- BMI must be equal to or lower than 30 (i.e. non-obese) or 32 for Maori/Pacific Islanders. Increased body fat percentage tends to make interoception more difficult [41];
- Regular levels of physical activity: at least 150 min of moderate-intensity aerobic physical activity throughout the week or least 75 min of vigorous-intensity aerobic physical activity or an equivalent combination of moderate- and vigorous-intensity activity (based on the *World Health Organization* guidelines). Regular exercise influences autonomic tone [42, 43], which is able to improve interoceptive accuracy as assessed by heartbeat perception [44–46];
- Normal or corrected vision;
- Normal or corrected hearing;
- Right-handed (left vs. right-handed people respond differently on RT tasks; see for example [47]; plus, this criterion removes the need for bilateral counterbalancing);
- Availability for the entire study period, including attendance at two laboratory sessions (one in May/June 2019, one in July/August 2019, with an 8-week interval between sessions).

#### Exclusion criteria

- Poor physical health (including gastrointestinal disorders, heart disease, kidney disease, respiratory disease);
- Poor psychological, psychiatric, or neurological health (e.g. alexithymia, schizophrenia, depression, anxiety, brain injury);
- Somatosensory amplification (score on the SSAS > 35);
- In pregnancy;
- Formal dance or drama training (see [48]);
- Formal musical training (see [49]);
- Formal meditation or mindfulness training (> 4 weeks consecutively over the past year) (see [7]).

Besides the case of pregnancy, which will likely affect physiological measurement, gender will not be part of the screening, despite men tending to outperform women in interoceptive sensitivity tasks (e.g. cardiac activity detection), due to the likely explanation for this difference being varying BMI across the sexes (see [50]).

### Randomisation

Each participant will be assigned a number ID in order of recruitment into the study. Once the required sample size has been reached and scheduling completed for laboratory attendance, these numbers will be entered into an online random number generator (http://www.randomization.com/) to allocate these numbers into one of the three blocks (cardioception group; gastroception group; control group). The allocation ratio for the groups will be 1:1:1.

Other randomisation pertains to counterbalancing over trials and blocks of trials. This will be undertaken to control for ordering effects and will be achieved using the standard functions of the stimulus presentation software programme *SuperLab (Version 6, Cedrus)*.

Participants will be told of their allocation immediately after the completion of baseline data collection. Concealment will be ensured using sealed notes provided during this first laboratory visit, within the process described below (see the “Confidentiality and single blinding” section).

### Participant retention

The likelihood of drop-out from the project will be reduced by offering compensation (supermarket vouchers) for the time commitment to the project, in addition to the attraction of meditation training, maintaining regular contact either by face-to-face (laboratory visits) or online monitoring (on *Qualtric*s), offering flexibility to participants on laboratory scheduling, and limiting the sample to those who are likely to be already on-site (either the University campus or Hospital, which are interconnected buildings).

### Study setting/location

Given the need to undertake laboratory tasks and the use of physiological measures, coupled with the local sampling possibility, all in-person data collection will take place in the Centre for Translational Physiology, University of Otago Wellington (UOW).

### The intervention

For those allocated to the experimental groups, an intervention involving one of two interoceptive practices will begin immediately after completing baseline measurements in the laboratory. Data will be collected at baseline and again 8 weeks later (a time scale based on a previous study of body awareness and interoceptive accuracy [7]).

Over the 8-week period, participants from each of the experimental groups will be instructed to log on to *Qualtrics* 6 days per week to record their responses to several qualitative questions on their everyday emotional experiences, and then to attend to their assigned body area for 20 min (2 × 10 min) after responding to these same questions (see Additional file 1 for the wording presented to the three groups). The 20-min period will be timed automatically with full task instructions provided. Participants will be requested to do this task at a set time each evening. All log-ins and time spent on the site will also be recorded and time stamped automatically, which will help with adherence, create a sense of connection for participants throughout the study period (in the absence of a bespoke class or programme to attend in person, participants will at least know their online attendance is monitored), and ensure that the task proceeds similarly for all participants. Adherence will also be encouraged by contacting all participants at the mid-point (i.e. 4 weeks after baseline) to check for problems or difficulties in adhering to the intervention instructions.

The instructions for cardioception will be to focus on the inside of one’s chest area without using non-interoceptive cues, throughout the daily 20-min period. These participants will be provided with an image of a human body with a highlighted chest area, which displays the target area for interoceptive focus i.e. (a limited area around the heart's location). The instructions for gastroception will be to focus on sensations in the lower abdomen (i.e. the dominant locus of gastrointestinal structures and activity). These participants will again be provided with an image of a human body, but with a highlighted lower abdominal area, displaying the target area for interoceptive focus. Importantly, this does not, then, incorporate the mouth and—in particular—the oesophagus, which overlaps with the location of the heart and would, therefore, be confounding as a focal point. Given the dominance of the lower abdomen for gut volume/activity, there is arguably relatively little *detectable* activity throughout much of the mouth/oesophagus unless a participant is experiencing gastric reflux, vomiting, or other pain, or is eating/has just eaten, which the sample screening aims to preclude in any case.

All participants from the experimental groups will be required to not undertake any additional meditation outside of the intervention, during the 8 weeks.

### Rationale for the control group

The control group participants will differ from the experimental groups only by their not undertaking meditation/interoception training during the 8 weeks. They will otherwise be “active”, i.e. they will be asked to log on to *Qualtrics* 6 days per week for 8 weeks to complete the self-reflection questionnaire. This will control for any effect of self-reflection or regular engagement in a task; it will measure any effect of interoceptive training in general, as well as any effect of each specific form of interoceptive training. The interoceptive groups together will also act as their own control for a merely general effect of interoception. If they choose to receive them, controls will be provided with the online meditation instructions and task once their participation is complete.

### Data collection procedures

#### Pre-laboratory

Prior to lab attendance at baseline and follow-up, participants will provide online (via *Qualtrics*) self-report measures on emotion regulation and health. The self-report tools to be used are the *Difficulties in Emotion Regulation Scale*, the *Emotion Reactivity Scale*, and *SF-36* sub-scales (outlined below in the “Descriptions of self-report tools for outcome measures (emotion regulation and interoceptive sensibility)” section). On an on-screen image of a human body, participants will also click to indicate the location(s) where they most tend to detect emotional feeling. If they do not detect emotional feeling in any part of the body, then no response is required. This is intended to identify the dominant areas of the body prior to entry into the study.

Other pre-task procedures will include providing instructions to participants stating that during the 4 h prior to their arrival they should not consume a heavy meal (but are adequately sated by the time of arrival in order to avoid distracting or facilitating hunger pains) or items containing caffeine or nicotine, or a large quantity of water (i.e. just maintain usual drinking amounts to prevent differences between participants in body awareness during the laboratory visit, such as awareness of the stomach or bladder), nor should they have consumed alcohol in the previous 24 h. In advance of attending, each participant will be asked (using the *SF-36*) if they have been experiencing any pain over the previous 4 weeks (which may interfere with their performance or that may prove distracting).

#### During laboratory visits for each data point

All participants will have the chance to use the toilet upon arrival at the laboratory. All participants will also be offered a half cup of water in order to attempt to equalise current levels of water intake in combination with the pre-attendance requirements (i.e. those who are less sated will be more likely to take up the offer). The pre-task procedures will be checked; participants will be rescheduled if necessary (such as if caffeine had been consumed). Mobile phones will be switched off prior to the start of experimentation.

Participants will undertake the spatial cueing task followed by the stop/signal task, then by a measure of interoception involving heart beat estimation, all on a laptop computer. This will be followed by further tests of interoception, including a water load test and a self-report tool. All tasks will take place in a physiology laboratory (with light attenuation on the first two tasks).

Heart activity will be measured using ECG throughout all computer tasks. This is commonly found in emotion studies as an objective indicator of affective changes. Heart rate variability (HRV) is measured by beat-to-beat changes in heart rate [51], where greater HRV is an indication of adaptability and has been associated with inhibitory processing (e.g. [52–54]). Rapid heart rate deceleration has been established to occur following the presentation of external stimuli [55, 56], but which is more marked when viewing emotionally negative stimuli (see [57, 58]). By taking continuous ECG measurements, this deceleration can be recorded and linked to the presentation of individual stimuli. In order to link the ECG with the stimulus presentation software, an audible tone will be the first event in each task, played through headphones with a microphone inserted into a speaker. The microphone will be plugged into the acquisition and analysis system *PowerLab* 16SP (*ADinstruments*). *SuperLab* will be set up to track the cumulative time (in milliseconds) throughout trials, so that the initial tone will then be the reference point for all other measurements in both *SuperLab* and *PowerLab* (using *Labchart* software, *ADinstruments*). Because the sound is played through headphones before each participant begins, this will have no relevance to any other tone played during the stop/signal or the Schandry task (the spatial cueing task involves no tones in its procedures). The magnitude of the heart rate deceleration (and “heart rate variability” more generally) can then be used as an objective physiological indicator of emotional responses.

In addition, constant measurement of heart rate will permit control of the influence of the systole heartbeat phase on responses to threat/fear stimuli (i.e. compared with diastole, responses to such stimuli during systole lead to greater arousal and are likely to be quicker [59]). This has the potential to create unmeasured variability in the data if stimuli are presented at varying heart phases.

Participants may attend with loose-fitting clothing, or a scrubs top will be supplied; this is to allow easy application of the ECG electrodes (which will be self-applied according to given instructions). A 3-lead ECG will be recorded with surface Ag/AgCl electrodes on the chest in standard configuration (*ADinstruments* ECG, NZ). *PowerLab* will be set at a sampling rate of 1 k/s, and with a range of 100 mv.

Participants will move to a seated position in an enclosed booth to prevent visual distractions. They next rest their head in a chin rest to prevent overt orienting, facing a computer screen (*Apple Macbook Pro* 15-inch laptop screen) at a horizontal distance of 45 cm from eye position and access a mouse to the right-hand side. To ensure the comfort of each participant, an adjustable height desk will be used.

In order to be able to answer any questions and, insofar as possible, to monitor adherence to the task instructions, the researcher will be seated at the far side of the laboratory room throughout the tasks, but unable to see/observe the participant.

#### Spatial cueing task

This will address RQ1 and RQ2. To address RQ1, an emotional cross-modal^1^ spatial cueing task will be used to record RTs to emotional stimuli (the *International Affective Picture System*, or IAPS; the *Nencki Affective Picture System*, or NAPS) when detecting activity at a location. The IAPS [60] is a well-established set of emotional stimuli, reliably inducing affect with short presentation duration [61], and without habituation during repeated exposure to same-valence images [62, 63]. The NAPS is a more recently developed, less widely used, but still validated visual stimulus set [64] using the *Self-Assessment Manikin* (SAM), which was also used for standardising ratings of arousal and valence in the IAPS.

The IAPS stimuli have been shown to elicit physiological responses, with one study [65] finding heart rate variability responses with images rated at arousal levels of ≥ 4.5 (based on the scale for SAM, 1–5, where in this case 5 was the highest level of arousal). If erotic imagery is excluded, negative emotional images that produce defensive responses, such as pictures of human or animal attack, tend to be the most highly arousing [66], eliciting startle blink responses, EMG responses, heart rate deceleration, and skin conductance in both men and women [67]. Based on findings such as these, and with the project’s overall focus on suicide prevention, it makes sense to focus on negative arousal.

A modified version of Thomas et al.’s [68] spatial cuing task will be used. The original study measured “interpersonal body representation” via responses to exogenous visual cues that shared a known location with tactile stimulation. Specifically, this used brief flashes of light on different locations of a projected model. Using a solenoid, a tactile stimulus was then applied to the same or different body part of the participant. Participants responded verbally as quickly (RT) and accurately (error rate) as possible. The findings from Thomas et al. indicated that valid cues resulted in faster RTs and fewer errors by contrast with invalid cues, suggesting that a cue in one mode can be effective for target identification in another mode. However, this was found only in the anatomical congruence condition (i.e. my right side is your right side), not the specular (i.e. my right side is your left side), which suggests a cross-modal relationship mediated by empathy (consistent with findings on “mirror neurons”, e.g. [69]).

In the modified version of the task to be used here, the RT to cues will be treated as indicators of a previously *unknown* location of emotional feeling to which participants have responded. Participants will be requested to indicate emotional signal detection upon presentation of an emotional stimulus, which follows a visually cued location on a computer screen (a seated figure with a highlighted area of the body). Faster RTs will be more likely if there is in fact a detected bodily location that is accurately cued. Alternatively, slower RTs will be more likely if there is a detected bodily location that *could be* (cross-modally) distracted from by the presentation of a divergent visual cue.

Consideration needs to be given to possible “noise” generated by these methods. Firstly, participants have been shown to have faster responses to a visual cue presented on the same side as the responding hand, which has been interpreted as a cross-modal cueing effect (see [70]). However, this is not always considered in relation to the well-established “Simon Effect” [71], which is where RTs are faster due to a primed motor response to the target rather than an attentional cross-modal effect. For this reason, the “interpersonal body representation” paradigm appears to be an advance on other spatial cueing approaches due to the identification of anatomical versus specular differences: the “Simon Effect” appeared to have been overridden by the social nature of the cue. Plus, the cues used in the current study will always be presented centrally, with no bilateral bias.

Other possible “noise” includes:

*The effect of overt bilateral orienting*. This is essentially a cueing effect since it cuts down possible attentional foci by 50% at the point of stimulus onset. The stimulus, or key details within a stimulus, may obviously be on one side of a screen or the other. For this reason, central fixation in visual cueing tasks is necessary to prevent variation across a sample in this respect.

*Lack of luminance*. In all visual cues and stimuli, isoluminant colour changes [72], isoluminant onset cues [73], or sudden onset stimuli that do not involve a luminance change do not capture attention exogenously [74]. For this reason, standardised presentations of visual stimuli with the validated ability to capture exogenous attention should be used.

*The effect of sudden onset only*. There can be ambiguity between specifically affective salience and the more general effect of the sudden onset of any physical stimulus [75]. For this reason, blocks of trials must include neutral stimuli as well as emotional stimuli.

*Creating expectations*. In *exogenous* attention tasks, the cues may become task-relevant (i.e. predictive of a target stimulus and hence *endogenous*) [76]. In exogenous tasks, attention is *drawn to* something, whereas in endogenous trials, attention is *directed at* something with an expectation of an event (e.g. the difference between being woken in the night by a fire alarm and waiting for the school dinner bell to sound). To address this, the frequency of valid cues should, ideally, not depart from 50% of total trials (i.e. equal to chance), with suggestions in the literature (see [77]) that > 70% of overall trials may be predictive of a target (i.e. endogenous). In the emotional spatial cueing task, cues often predict the target location in 70% or more of the trials (see, for example [78]), which may facilitate task relevance.

**Fig. 2:**
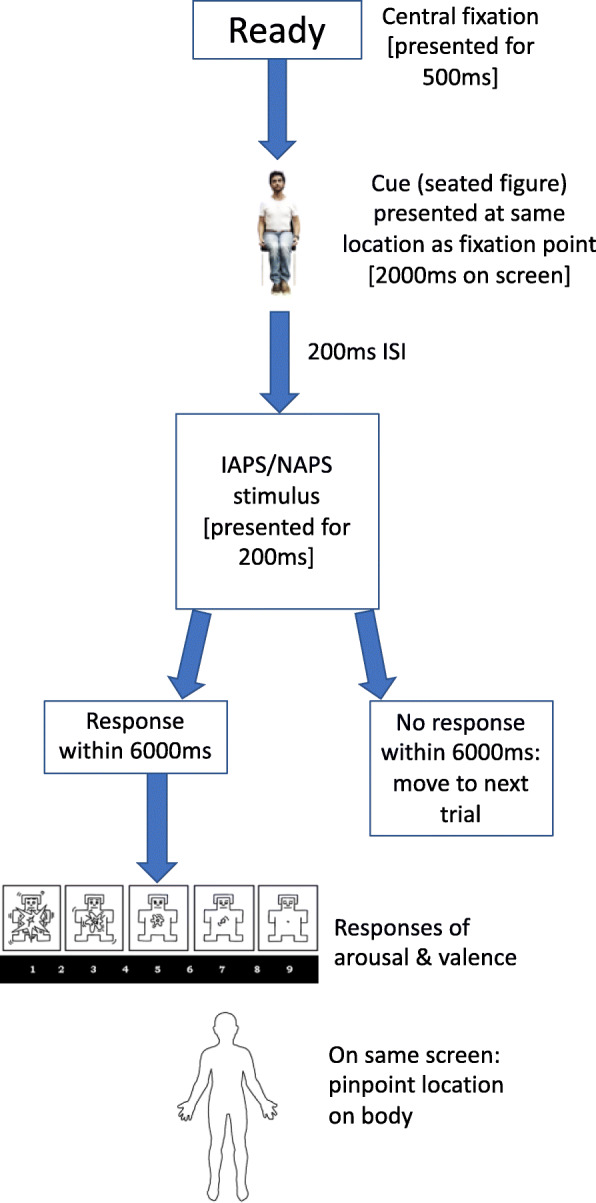
Outline of the spatial cueing task

Based on the foregoing, the spatial cueing task procedure will continue as follows:

- A practice block of 16 trials will be completed (using 8 neutral emotional stimuli, involving each of the cued locations, and neutral cues).
- In each trial, participants fixate on a word (“Ready”) at the centre of the screen for 500 ms prior to cue onset (Fig. 2).
- The cue (a seated male figure) will be presented for 2000 ms in the middle of the screen, either with one of three locations highlighted at random using a red, highly luminous highlighted rectangular area (see [68, 79]), or no location highlighted. The three locations will be the chest, lower abdomen, or shins (pressed together in the image to form a single area). All highlighted areas will be the same size, colour, and luminescence. The image will be large (345 × 715 pixels), and the highlighted area as luminescent as possible within the limitations of the package used to create it (*Microsoft Office Word, version* 16.23).
- The emotional stimuli (selected IAPS and NAPS images; see Additional file 2) will be either 600 × 800 pixels or 800 × 600 (i.e. landscape or portrait), all at a resolution of 72 pixels/inch. The stimuli will be delivered at an inter-stimulus interval (ISI) of 200 ms, remaining for 200 ms (changes in levels of arousal do not increase significantly beyond 150 ms presentation duration [61]). With picture stimuli being presented to elicit exogenous attention, a mixture of neutral and negative emotional images will be presented to control for the effect of sudden onset of a stimulus. Further, since negative stimuli tend to reliably create the highest level of physiological arousal in men and women, this will be important for creating detectable changes in the body. Again, to ensure that the cues do not become task-relevant (i.e. predictive of a target stimulus and hence endogenous), the frequency of valid cues will be kept to below 70% of trials. Although it is not known in advance what a “valid” cue is in this bespoke variant of the spatial cueing task, for the purposes of setting up the cues these are limited to the chest and lower abdomen areas (interoceptive/visceroceptive areas), each with 31% of “valid” cues, which remains within the standard limits (i.e. 62% in total). A further 10% of trials will be “invalid”, i.e. the shins (a non-interoceptive/visceroceptive area).
- In 15% of trials, there will be no emotional stimulus following a cue (catch trials); plus, in a further 14% of trials, there will be a neutral cue (no cue areas highlighted)^2^.
- Non-catch “valid”, “invalid”, and neutral trials will be further split into calculated proportions of negative and emotionally neutral target stimuli. In total, there will be 65% negative and 20% neutral stimuli (along with the 15% catch trials).
- Participants are required to give a speeded response: a mouse click upon detection of a bodily change—anywhere in the body—but they will be instructed to be sure of detection (i.e. guesses are discouraged; see [77]). It is important to separate this initial response from the indication of a location in order to clearly disambiguate (i) no emotional feeling being detected and (ii) an emotional feeling being detected but when the location was not known. If no response is registered within 6000 ms, the next trial is presented.
- If a change was detected, then participants transition to another screen to indicate a location on a virtual body by mouse click, followed by an indication of the valence and intensity of the detected feeling (using the *Self-Assessment Manikin*; for a similar approach, see [19]). If a location was detected but it was not known where, this can be indicated by clicking outside of the body area. The time taken to give these further responses will assist with a likely longer refractory period for an embodied emotional response compared with, say, visual attendance. (See, for instance [80], where the mean cardiovascular recovery time following an emotional film stimulus—lasting up to 100 s in duration—was approximately 30 s. The estimated time per trial in the current spatial cueing task, including an inter-trial interval of 200 ms, is around 15 s, but where a picture stimulus will be presented and for a far shorter duration of 200 ms, which may then result in a shorter recovery period than a relatively lengthy film.) This process will be repeated throughout all blocks, where the cued response is exogenous.
- In total, there will be 6 main blocks, with a brief rest period between them, and 248 trials. Based on the percentages stated previously, this will result in 152 valid trials (76 lower abdomen; 76 chest), 26 invalid trials (shins), 37 catch trials, and 34 neutral cue trials (where no part of the body is highlighted). For each of the valid sets of trials, 58 emotional stimuli will be negative and 18 will be neutral. For invalid trials, 20 will be negative and 6 will be neutral. For neutral cue trials, 26 images will be negative and 8 will be neutral. For catch trials (no emotional stimulus), there will be 10 sets of each of the 3 bodily locations, with one set of 7 neutral locations (no areas cued on the image of a seated person).
- Finally, to control for order effects, blocks will be counterbalanced: one half of each of the groups (assigned at random) will complete blocks in the order 1–6. The other half will complete the blocks in reverse order.

#### Stop/signal task

The subsequent task addresses RQ3 and will use a second experimental paradigm involving the same experimental apparatus: a stop/signal task, a variant of the better-known go/no-go task. Whereas the latter requires participants to respond rapidly to a target stimulus (“go”) and withhold a response to a non-target stimulus (“no-go”), the stop-signal task usually requires infrequent withholding of a response when presented with a randomly presented “stop” signal following a choice task between frequent presentations of two “go” stimuli [81, 82]. Also, whilst “go/no-go” tasks have zero stimulus onset asynchrony (SOA), stop/signal tasks have variable SOA. Where the “stop signal delay” (the interval between stimulus presentation and stop signal presentation, i.e. the SOA) is short, participants can easily inhibit a response. With longer delays, participants are more likely to execute a response and, thereby, have failed to inhibit. The stop-signal task, then, represents a more difficult test of response inhibition than go/no-go tasks due to the increased likelihood of a pre-planned response being executed when there is more time for execution, in combination with the higher frequency of “go” versus “stop” signals.

The “Horse Race Model” [82–84] is a theoretical explanation for performance on the stop/signal task, where go and stop processes are deemed independent and “race” each other to their respective thresholds and, thereby, to one of two outcomes: a response or response inhibition. By varying the SOA (between the “go” choice stimuli and “stop” signal: termed “staircasing”), a time estimate for response inhibition can be calculated from the mean SOA required for a 50% success rate of response inhibition and the mean RT to Go stimuli. This is the “stop-signal reaction time” (SSRT). The current study will be based on a key study of *emotional* inhibition undertaken by Verbruggen and De Houwer [35].

These authors reported that when using an emotional target stimulus in a stop/signal task, compared with neutral stimuli, this resulted in slower RTs due to increased emotional activation (i.e. greater task interference from emotional stimuli). The current study will vary from their approach somewhat in that the Verbruggen and De Houwer also investigated differences in impact from varying valence and intensity of imagery (finding only an effect from the latter), whereas the current study seeks to compare levels of inhibition to arousing stimuli of *either* valence, and using neutral stimuli for similar reasons to the spatial cueing task (as a standard control for the sudden onset of a visual presentation). Using an array of valence scores aims to support a more general emotional effect (which the study on spatial cueing is not able to do given the need to maximise high arousal stimuli).

**Fig. 3:**
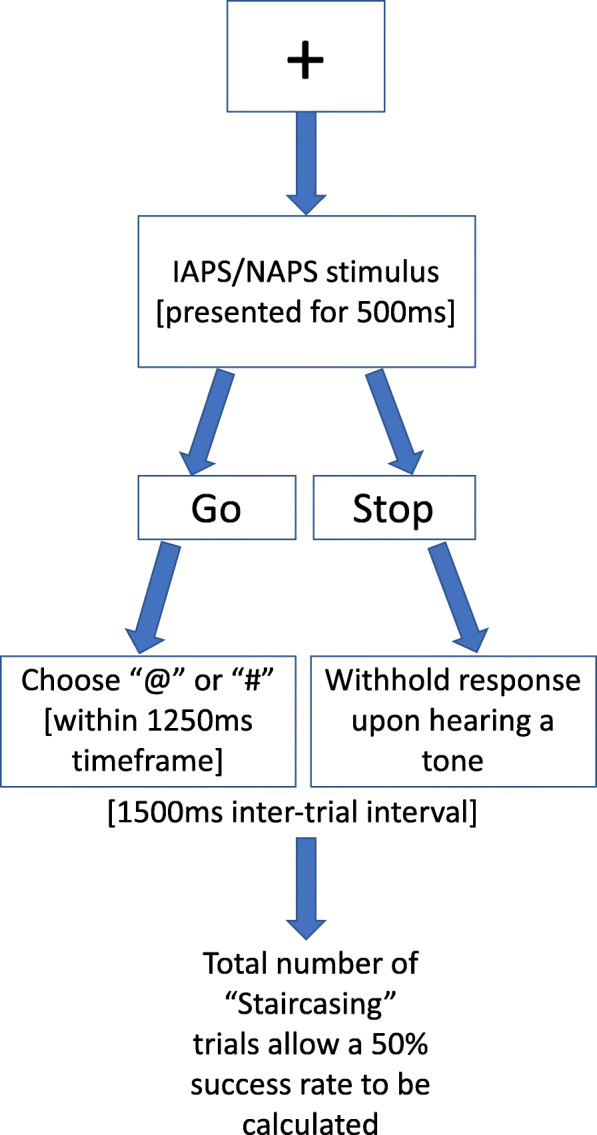
Outline of stop/signal task

The full procedure will run as follows:

- Instructions are provided on-screen for the practice block. This training block consists of 24 trials using 8 neutral IAPS/NAPS images, otherwise following the same process as for the experimental phase. For the experimental phase (8 blocks of 60 trials), 120 images will be used (40 positive, 40 negative, 40 neutral), with each image appearing four times. The set of 120 images (see Additional file 2) will be split into two halves, with half presented in even numbered trials, and the other half in odd numbered trials.
- For all blocks, participants first fixate on a cross at the centre of the screen for 500 ms (Fig. 3); IAPS/NAPS stimuli are then presented for another 500 ms.
- On “go” trials, participants must respond to the visual stimuli “#” or “@” presented at the centre of the screen, pressing a corresponding key as quickly and accurately as possible after presentation. These symbols will be reproduced as stickers on the keyboard to ensure participants make the required key press. Responses must be completed within 1250 ms before the next trial begins. After incorrect trials, this ISI begins following a 250 ms delay. The word “Incorrect” appears when there is an error, “Respond” if there is no response when there should be, and “Stop” where there is a response but should not be.
- On “stop” trials (indicated by a tone on headphones; 750 Hz, 50 dB, 100 ms; presented randomly in 30% of trials = 48 stop trials per valence set), participants must withhold a response to the visual stimulus. The stimulus onset asynchrony (SOA; the stop-signal delay) between the choice task stimulus and the auditory tone will begin at 250 ms, then vary by staircasing: a response on a “stop” trial results in a decrease in the SOA by 50 ms, successfully withholding a response on a “stop” trial results in an increase of 50 ms.
- Participants will be informed of this auto-adjustment in staircasing, to discourage their anticipation of the “stop” signal and to focus only on rapidly responding to the symbols (as emphasised in Verbruggen and De Houwer).
- If in the unlikely event a participant presses before the tone, a neutral message of “Next trial” appears on-screen in order to ensure that random, rapid key pressing is discouraged whilst at the same time not penalising genuine attempts to respond rapidly. (This is a bespoke part of the design, not taken from Verbruggen and De Houwer.)
- The time between trials will be 1500 ms.
- At the end of each block, performance feedback is provided to participants: the number of correct “go” responses (i.e. pressing the correct symbol), number of incorrect “go” responses (i.e. pressing the wrong symbol), number of incorrect “go” non-responses (i.e. too slow), number of correct “stop” non-responses (i.e. withholding a response when hearing a tone), and number of incorrect “stop” responses (i.e. responding in the presence of a tone). Trials where “Next trial” appears on-screen are not included in this tally.
- There will be a 30-s gap between blocks.

#### Counterbalancing

For half of the participants in each group (assigned at random), the blocks will be presented in the order 1–8; the order will be 8–1 in the other half.

#### Measuring interoception

Towards the end of each laboratory visit, measures of interoception will be taken. Interoceptive sensitivity will be assessed using two different methods.

#### Schandry heart tracking task [85]

This will be undertaken by all participants, again using *SuperLab* and *Labchart* with *PowerLab*. This entails a participant counting their heart beats within set time periods (a training interval of 25 s, followed by four experimental intervals of 25, 35, 45, and 55 s presented in random order) without taking their own pulse or using any other non-visceroceptive cues (e.g. hearing their heart beat, watching a part of their body pulse in tandem with heart beat) at the same time as the actual heart rate is measured using ECG. The onset of each time period is signalled by a single tone played through headphones, with the offset of each period signalled by a double tone (200 ms gap between two single tones). Participants will then type in their estimated number of heart beats. Each counting period will be separated by a 30-s interval. For ECG recordings, the single and double tones will be used as reference points to permit the calculation of difference between actual heart rate and perceived heart rate, as a measure of interoceptive sensitivity (accuracy).

#### Water load test [86]

After disconnecting from the ECG and computer, the water load test will be undertaken by all participants. This has two stages: the first stage entails participants’ self-assessment of having reached a state of satiation following drinking water from a 5-L glass container through a 4.8-mm clear PVC 1 m tube. The glass containers will be covered to prevent participants from knowing the volume of water in each, to give a sense of a practically unlimited supply of water. Unknown to participants, each flask contains no more than 1.5 L of water. This limitation on the amount of water prevents the risk of water intoxication, in combination with the screening measures in place regarding kidney health. Further, in addition to the previous use of this task in published, peer-reviewed research, this component was deemed safe from consultation with an endocrinologist and following the University ethics process. Further, this was confirmed by a second physician (who will provide oversight of the project in general).

The second stage entails drinking water from an identical set of apparatus until reaching a sense of fullness. In the original paradigm, each participant was not informed about this subsequent stage until after the first stage was complete. However, given that this measure will be repeated over two data points, participants will be informed about the second stage prior to the onset of the task to ensure consistency across data points^3^. Each stage of the task is limited to 5 min. The amount of water consumed at each stage—measured by weight differences of the containers between pre- and post-consumption of water—is then used to calculate the participant’s interoceptive sensitivity to gastric sensations. Due to the fact that having a full stomach of water would likely confound measurement during the previous tasks (not least due to relative discomfort), this measure of interoception will be taken towards the end of each laboratory visit. Also, running the water load test at the end (approximately 2 h into their visit) also helps to ensure greater parity between participants in terms of prior water intake.

Finally, each participant will complete a brief self-report questionnaire to measure interoceptive sensibility (The *Multidimensional Assessment of Interoceptive Awareness* or MAIA [87]). This is outlined in the next section.

In undertaking measures of sensitivity and sensibility, this also allows the calculation of metacognitive awareness; in this way, all three forms of interoception can be measured [88].

After the 8-week intervention period, participants will attend the follow-up, involving repetition of all procedures outlined above. Participants will be reminded at the start of the second visit that all procedures will be identical and to perform them as at baseline.

## Descriptions of self-report tools for outcome measures (emotion regulation and interoceptive sensibility)

The *Difficulties in Emotion Regulation Scale* (DERS) consists of 36 items (reduced to 18 in a validated short version [89]) in six subscales [90]: (i) non-acceptance of emotional responses, (ii) difficulties engaging in goal-directed behaviour, (iii) impulse control difficulties, (iv) lack of emotional awareness, (v) limited access to emotion regulation strategies once an emotion is in process, and (vi) lack of emotional clarity. (See, for example [91] for information on the scale’s psychometric properties.) A key rationale for using a tool such as this is to assess how the measures taken in the laboratory (i.e. at an early stage during an emotional reaction) may relate to later stage emotion regulation. For instance, decreased RTs in the spatial cueing task may indicate increased access to emotional sensations and which may relate to emotion regulation.

The *Emotion Reactivity Scale* (ERS) consists of 21 items in three subscales (i) the sensitivity to emotion (e.g. “Other people tell me I’m overreacting”), (ii) intensity (e.g. “When I experience emotions, I feel them very strongly/intensely”), and (iii) persistence (e.g. “When something happens that upsets me, it’s all I can think about for a long time”). (For information on the scale’s psychometric properties, see [92]). Regarding this measure, according to some theorists, the primary reason given for the decision to attempt suicide is to escape from aversive and intolerable emotion [93–96], which may result from increased emotional reactivity associated with some psychological disorders [92]. The rationale for the use of the ERS in the current trial is the same as for the DERS.

The *SF-36* is a short-form health questionnaire. For the purposes of the current project, several sub-scales have been chosen to track physical and mental wellbeing over time: Physical Functioning; Emotional Well-being; Pain; General Health (see https://www.rand.org/health-care/surveys_tools/mos/36-item-short-form.html; RAND Corporation).

The *Multidimensional Assessment of Interoceptive Awareness* [87] (MAIA) is a 32-item questionnaire involving 8 scales: (i) Noticing: awareness of uncomfortable, comfortable, and neutral body sensations; (ii) Not-Distracting: tendency not to ignore or distract oneself from sensations of pain or discomfort; (iii) Not-Worrying: tendency not to worry or experience emotional distress with sensations of pain or discomfort; (iv) Attention Regulation: ability to sustain and control attention to body sensations; (v) Emotional Awareness: awareness of the connection between body sensations and emotional states; (vi) Self-Regulation: ability to regulate distress by attention to body sensations; (vii) Body Listening: active listening to the body for insight; and (viii) Trusting: experience of one’s body as safe and trustworthy. One advantage of using the MAIA is that it is able to differentiate between interoceptive sensibility that is linked to “somatization” (a problematic hyper-awareness of the body) and interoceptive sensibility linked to well-being.

## Variables

### Predictor variables (both tasks)

- Group membership ([interoception groups] vs. [control];
- Data point/time (baseline vs. follow-up);
- Interoceptive accuracy/sensitivity (continuous measure);
- Interoceptive sensibility (continuous measure);
- Interoceptive metacognitive awareness (continuous measure);
- Heart phase ([systole] vs. [diastole]);
- Valence of emotional stimuli ([positive: *stop/signal task only*] vs. [negative] vs. [neutral]).

### Predictor variables (spatial cueing task only)

- Presence/absence of emotional stimulus (i.e. “catch” trials);
- Cued position on body in the spatial cueing task ([shins] vs. [chest] vs. [lower abdomen]; or [a part of the body cued] vs. [no part of the body cued]);

### Dependent variables (both tasks)

- *Primary outcome*: RTs;
- Scores on self-report measures;
- Heart rate variability, heart rate deceleration.

### Dependent variables (spatial cueing task only)

- Frequency of response to emotional stimuli;
- Intensity of experience (continuous);
- Valence reported ([positive] vs. [negative] vs. [neutral]);
- Location of felt experience (point indicated on a human model; to be coded into a categorical measure).

## Hypotheses

Based on the foregoing detail, the hypotheses for the studies are as follows:

### Spatial cueing task

1. One of the interoception groups will show significantly faster reaction times (RTs) and greater response frequency to emotional stimuli regardless of cue type compared with each of the other groups after baseline; plus, there will be a greater change between baseline and follow-up. Each of the interoception groups separately and combined will show significant differences from the control group.
2. One set of cues for the same group (i.e. the interoception group in (1)) will facilitate significantly faster RTs and greater response frequency. This set will match the target area of the body for that group (i.e. a chest cue for the cardioception group; a lower abdomen cue for the gastroception group). The “matching” cue for the other interoception group will not facilitate faster RTs or greater response frequency. If any cue facilitates faster RTs or greater response frequency for the other groups, it will be the same cue that facilitates for the interoception group in (1).
3. This same group will display greater within-group coherence in the location of the body indicated to be active during trials compared with the other groups after baseline. It will also show the greatest change between baseline and follow-up compared with the other groups.
4. This same group will report greater intensity compared with the other groups after baseline. It will also show the greatest change between baseline and follow-up compared with the other groups. The interoception groups combined will report greater intensity than the control group.
5. This same group will show greater heart rate deceleration after baseline following the presentation of emotional stimuli compared with the other groups; both interoceptive groups will show greater heart rate deceleration than the control group.

### Stop/signal task

6. This same group will show significantly slower RTs on the stop-signal task after baseline and a greater change in RTs between baseline and follow-up compared with the other groups, but with each of the interoceptive groups separately and combined showing significantly slower RTs compared with the control group.

### Hypotheses on interoception and emotion regulation:

7. Both interoceptive groups will show increases in interoceptive sensitivity (measured by the Schandry task and water load test), sensibility (measured by the MAIA), and metacognitive awareness over the study period, but there will be no significant differences between each other either at baseline or between baseline and follow-up. They will each be significantly different from the control group on these measures. Further, the cardioception group will show the greatest improvement on the Schandry task, and the gastroception group will show the greatest improvement on the water load test.
8. The two experimental groups will each show significant improvements over the control group on the *DERS*, the *ERS*, and the *SF-36* (emotional well-being sub-scale) scores between baseline and follow-up, with the same group as in (1) showing greater improvements compared with both of the other groups.

## Data analysis

Generalised Estimating Equations (GEE) will be used to analyse the two data points, including variable interactions. GEE can account for repeated measures as well as the potential problem that the primary outcome variable (RT) may not be normally distributed (although it will be continuous). Using GEE, the effects of the predictors on RT can be modelled under different circumstances, and the interrelationships between predictors can also be investigated. Significance testing will be undertaken at the *p* < 0.05 level.

## Discussion

To our knowledge, this will be the first project to investigate whether emotion presents as a localised phenomenon in the body and, further, whether awareness of an active location can be trained, with a view to improving emotion regulation. The extension of these empirical studies to encompass a conceptual analysis of how the findings may relate to theories of suicide will be additionally novel.

Although there is a low chance of harm coming to any of the participants from taking part in the project, particularly due to the decision to recruit only healthy participants at this stage, there will be several processes of reducing risk. Firstly, given that participants will be responding to questionnaires containing reference to emotional sensitivity and health status, there is a small possibility of a negative reaction to these. Further, during the intervention, some participants will be asked to attend to their physiological activity, which is consistent with some meditative practices. Although there are no studies reporting any complications with interoception specifically, the meditative overlap may lead to some of the difficulties that have occasionally been reported with some practices [97]. Also, during the laboratory tasks, participants will be exposed to standardised emotional stimuli designed to elicit strong emotional responses.

As a response to these risks, the screening procedures are in place to ensure that people experiencing psychological/psychiatric difficulties will be excluded from participation. This aims to reduce the possibility of a pre-existing condition that lowers the threshold for possible anxiety or stress in relation to the emotional stimuli, questionnaires, or undertaking meditation. Further, the contact email address for the lead researcher will be available to all participants, who will be encouraged to raise any concerns, bearing in mind that they will be undertaking a daily interoceptive practice so will have regular reminders of this support. Similarly, at various points throughout each laboratory session, participants will be asked if they have any concerns regarding the emotional content of any of the stimuli, or any other aspect of the procedures. Ultimately, any concerns raised by participants will be referred to the supervisory team, two of whom are registered clinicians in psychology/psychiatry. Further, a medical doctor linked to the project can provide guidance on physical health.

One key source of risk, identified at the time of developing the theoretical framework, will also be addressed using screening. Much of the literature on emotion regulation emphasises the critical importance of inhibition to prevent the individual from becoming overwhelmed (e.g. [98]). One of the more extreme cases of apparent *disinhibition* in relation to emotion and the body is “somatosensory amplification” and related constructs, such as “somatising”, “catastrophising”, and “hypochondria”. These are related to anxiety disorders and others, suggesting that increased interoception is not a realistic option for those affected by amplification of bodily signals. Hence, it would be important to exclude those experiencing amplification from participating in the project. Screening will be undertaken using the SSAS. The overall score on the SSAS is simply a sum of scores for each item (i.e. a maximum of 50 from 10 questions using a 5-point scale).

Whilst there is no established cut-off for high somatosensory amplification in the SSAS, some studies have indicated a score of around 30 to be potentially problematic. For example, Nakao and Barsky [99] indicate that the averages for healthy men and women are slightly below 30, with non-healthy averages somewhat over 30, including cross-culturally (i.e. Japan, Turkey, France, the USA). The standard deviations for these studies show a degree of overlap between healthy and hypochondriac groups, meaning that an unspecified number of healthy people will likely score over 30. In order to reflect these concerns, in the current protocol, the limit has been set at a maximum score of 35. Whilst higher than the suggestions in the literature, this will ensure that healthy individuals are not excluded whilst also ensuring that amplifiers at the higher end of the scale will be excluded. In combination with the other screening measures—given the aim to ensure that ultimately only healthy individuals will be recruited—this will limit any potential negative impact indicated by an SSAS score > 30.

However, somatosensory amplification may be an issue only for those who engage in the negative appraisal of bodily changes, rather than increased body awareness itself being problematic: physiological activation has been found to be no higher in those with somatosensory amplification than those without it (e.g. [100]). To disinhibit emotional sensations, then, does not necessitate catastrophe, unless one is already prone to cognitive “catastrophising”. Hence, increased interoceptive sensitivity may not, per se, present a risk to participants, although we have erred on the side of caution.

The findings of the studies will be disseminated by journal article publications, public availability of a PhD thesis, and by conference presentations.

## Limitations

There are several limitations of the current design.

For the spatial cueing task, attempts have been made to make all trials comparable in terms of timings, regardless of response. However, on trials where no emotion is detected, there will be a maximum of 6 s to respond before the next trial; but on trials where there is a response, the timing may be longer if a participant takes time to consider their response, or shorter if a participant becomes fatigued and aims to rush through the trials by clicking in response to all stimuli. In order to prevent the former problem, participants will be instructed to respond as quickly as possible (to reduce the effect of cognitive appraisal). In relation to the latter problem, the use of catch trials and emotionally neutral trials will indicate this trend in a participant’s data (which can then be excluded from analysis).

Secondly, the effect of a negative stimulus in the spatial cueing task may take time to dissipate (i.e. the refractory period). Hence, there may be carry-over from one trial to the next. This limitation is addressed to a degree by having a mixture of negative and neutral stimuli, plus varied levels of standard arousal (i.e. based on previous studies of rated responses to images on the IAPS and NAPS). Plus, the time to respond in full to each stimulus before experiencing the next stimulus may approach the end of a refractory period in any case, and between blocks, there will be self-directed rest periods.

Thirdly, there is a possibility that presenting the same set of emotional stimuli over the two data points—in both main tasks—will result in habituation across the two presentations. There is, however, a low probability of this given the brief presentation of each stimulus, plus an 8-week gap between data points, and the use of the IAPS, which has been shown to resist habituation.

There is likely to be some degree of self-selection bias in the sample, given the wholly voluntary nature of the recruitment, both in terms of consenting to participate and trial completion.

Finally, as a preliminary trial, there will only be recruitment of a non-suicidal sample. This will not itself allow findings on emotion regulation to be directly related to suicidality.

## Trial status

Protocol version 1.

Date: 20 March 2019.

Date submitted (original submission): 12 May.

Recruitment end: 13 May.

Date of trial completion: August 2019.

N.B. the ANZCTR specifies that a prospective randomised trial is one where the registration date occurs prior to the date of first participant enrolment. Further, it defines the date of first participant enrolment as the date upon which the first participant is randomised.

## Protocol amendments

Substantive changes to the protocol will be conveyed to the ANZCTR and the University of Otago Human Ethics Committee. These would include any changes to the sample characteristics, the intervention, active control processes, laboratory tasks, or the time scale of the project that may impact on participant experience or on the outcome measurements.

## Supplementary information

**Additional file 1.** Daily task instructions

**Additional file 2.** Stimuli selected for the tasks

**Additional file 3.** Consent form

## Data Availability

The quantitative datasets used and/or analysed during the current study are available from the corresponding author on reasonable request. The qualitative datasets generated and/or analysed during the current study are not publicly available due to potentially containing identifiable information.

## Abbreviations

DERS: Difficulties in Emotion Regulation Scale
ECG: Electrocardiogram
ERS: Emotion Reactivity Scale
GEE: Generalised Estimating Equations
HRV: Heart rate variability
IAPS: International Affective Picture System
ISI: Inter-stimulus interval
MAIA: Multidimensional Assessment of Interoceptive Awareness
NAPS: Nencki Affective Picture System
RT: Reaction time
SAM: Self-Assessment Manikin
SOA: Stimulus onset asynchrony
SSAS: Somatosensory Amplification Scale
SSRT: Stop-signal reaction time
UOW: University of Otago Wellington

## Footnotes

1 Across different modes of perception: the task involves a cue in one mode or modality (i.e. visual) but the response relates to another modality (i.e. a tactile perception), hence ‘cross-modal’.

2 N.B. Total > 100% due to rounding.

3 This decision followed communication with the lead author of the publication on which our use of the water load test is based.
