# Supplementary material for: Where is an emotion? Using targeted visceroception as a method of improving emotion regulation in healthy participants to inform suicide prevention initiatives: a randomised controlled trial
Source: Trials. 2020 Jul 14;21:642. doi: 10.1186/s13063-020-04479-9 (PMC7362633; doi:10.1186/s13063-020-04479-9)
Supplement: Supplementary file 1 — Additional file 1. Daily task instructions [file 13063_2020_4479_MOESM1_ESM.docx]

**Appendix 1 – Daily task instructions**

**Wording for experimental groups’ instructions**

*Introduction*

Having completed your first visit to the laboratory, you will now begin the daily task.

Each day, you will begin by answering several questions, followed by a simple meditation task, and a follow-up question. The questions encourage reflection on your emotional responses since your previous log-in. In this way, you can use these questions to document any experiences that have affected you personally. You should feel free to write openly, since your responses will be viewable only by the researchers.

The meditation task is a timed session, intended to give you a framework within which to develop skills in body focus. It will be unguided (i.e. no voice-over), with the emphasis on you to put into practice the instructions provided below.

You will complete this daily task 6 days per week, for eight weeks, followed by a second (and final) laboratory visit. For the duration of the study, please avoid doing more than this amount of meditation per day. At the 4-week midpoint, the lead researcher (Steven Davey) will be in touch (by Skype/Facetime, or email if you would prefer) to see how you are getting on and to answer any questions.

To support you throughout the project, the researcher will check-in regularly with this website to ensure the daily task is running smoothly and to encourage a sense of connection for the 8-week duration. Your efforts will not go unnoticed!

Please click the button below to continue.

[Next page]

When you are ready, please answer the following few questions as briefly or as comprehensively as you wish.

These will be asked every time you log in and are intended to capture any experiences you may have had since your last log-in. (N.B. if this is your first log-in, please respond in relation to any experiences over the last 24 hours.)

- Can you recall any experiences since your last log-in that you would describe as “emotional”?
- Please describe the bodily features of these experiences, indicating too whether the emotion involved was positive or negative, and the type of emotion (e.g. joy, anger).
- If there were several emotional experiences, did these share any bodily features? If so, what?
- Since your last log-in have you noticed any changes in your behaviour or thinking? If so, please describe these.

Thank you for answering these questions. Please click below to continue to the daily task.

*Today's 20-minute meditation session*

If this is your first session, please select “Read instructions” below. These can be accessed in each session as a reminder. N.B. it is advisable to read these instructions several times during the first few weeks.

Once you indicate that you are ready, the task will begin automatically. Please make sure you know what to do before you begin!

*Instructions*

*The environment*

The room where you do the session each day should be at a temperature that allows you to sit comfortably for 20 minutes. The lighting should be at a medium level: too brightly lit may make it difficult to relax, too dimly lit may make it too easy to relax (and fall asleep). Please also ensure there are no distractions, including food or drink, or any tabs open on your web browser that may distract you when viewing your screen. Please also ensure that you use a comfortable chair, and preferably one that will not move during the task (e.g. a dining room chair would be preferable to an office swivel chair).

*Body posture*

Please ensure you are sitting comfortably and able to maintain a stable, upright position, with a natural curve to your lower back. You can do this by leaning forward then bringing your shoulders back in an arc whilst keeping your lower back in a fixed position. Your head should be in an upright position, chin tucked in slightly, with your feet resting flat on the floor.

Once you are ready to settle into the meditation session, grip your left thumb with your right hand (as if shaking hands with yourself) and place your hands in your lap for the duration. Your eyes should be half open, and lowered at around a 45 degree angle (not at your screen). The mouth should be closed, breathing steadily through your nose. This will be the required posture for each session. You will have some additional time during each session to set up or check this posture.

*The focus*

Once the session begins, please give your full attention to your assigned body area (provided to you during your first laboratory visit). Please give your attention to deep within this physical area, to experience any sensations *inside* your body cavity. If you experience only the movements or sensations of the wall of your body cavity (i.e. the activity of your skeletal muscles) go yet further inside the body to the *organs and structures there*. Skeletal muscle activity is not the focus of the task.

*thinking* about what you are focussing on. Indeed, thinking obscures your attempt. The task involves pure observation. For instance, if you find yourself imagining the inside of your body, this would be thinking about - rather than purely observing - what is happening there. The act of focusing in the daily task is more similar to the attention you give, for instance, when tasting food or when threading a needle.

Tasting food does not involve thinking about the taste of food: you just taste it. The taste itself is not a thought. Threading a needle does not involve thinking about threading a needle: you just stare intently at the tiny opening of the needle and the slim filament at the end of the thread, slowly moving it millimetre by millimetre towards the needle’s opening. Thinking about threading the needle is not required (or helpful) for the act of threading a needle. In each example (tasting; threading) what is being focussed on consumes your attention: you get drawn into it. This is how the daily task should be done.

If your attention wanders, bring it straight back to the assigned area of your body the moment you regain awareness. For this practice to be effective, it is essential that there is genuine effort given throughout each session. It will likely be difficult at first, but results will come from your applied effort. At times you may feel tired or bored: this is completely normal. You may find yourself wanting to fidget or that your attention wanders. Maintaining attention for the 20 minute session will become easier over time; the more you try the easier the task will become. In the meantime, don’t be too hard on yourself. Although having to bring attention back again and again feels like failing the task, this is actually just a part of your learning to do it. Rather like lifting weights in the gym, if you continue to try despite faltering you will develop the required skill and strength to do the task. And - rather like lifting weights - the more you put into meditation, the greater the reward.

Use the first week for getting used to the change in your schedule, to trying to maintain focus during the timed session, becoming familiar with the required body posture, making sure you have a room that is reliably conducive to meditation, and to ensuring that the computer program runs reliably on your machine. The first week is, in general, an opportunity to make sure you know what you are supposed to do for the 8-week period, and to ask any questions you may have. By the end of the first week, you will have eased yourself gently into the task, and be ready to get the most out of the remaining weeks. If you experience difficulties in maintaining your attention and feel you need further support before the 4-week midpoint, please contact Steven Davey (davst397@student.otago.ac.nz).

*The structure of each session*

You do the same task each time you log in. Please try to do it at the same time each day so that it becomes a habit. Ideally, this should be in the evening, after dinner, so you are not distracted by hunger, long enough after dinner so you do not feel full, but not so long after dinner that it is nearly bedtime. Between 8pm and 10pm would be ideal, depending on your schedule. Those on shift-work may need to adjust these times accordingly. The session will be timed as two periods of approximately 10 minutes with an intervening 1-minute rest period to allow you to stretch. To remind you of where to focus, each period begins with the on-screen appearance of your assigned body area. As described, give your attention to deep within this body area and *only this area*. You can say to yourself “Here and nowhere else” to remind you of the need to focus attention on this one location only.

Meditation bells will sound to signal the beginning and end of each period, so you will not need to look at your screen. If you find the screen distracting, you may wish to switch it off once the bells have signalled the start of each period. If you do so, please be sure to switch it on again when hearing the bells signalling the end of the 10-minute period.

After reading and understanding these instructions, please adopt the body posture described and maintain this position for each 10-minute period, remaining still. When ready, click below to begin the task for today, which will then run automatically through to the end.

[Wording presented at set times in the following order, throughout the meditation session]

- The first period is about to begin. Give your full attention to the inside of the highlighted area of the body...[image of highlighted body area presented] “Here and nowhere else”.
- The first period begins now…
- Please lower your eyes until hearing the next bell...you may wish to switch off your screen until then.
- [Halfway point] A 1-minute break begins now...feel free to stretch then get ready for the second period.
- The second period of the session is about to begin. Give your full attention to the inside of the highlighted area of the body...[image of highlighted body area presented] “Here and nowhere else”.
- The second period begins now…
- Please lower your eyes until hearing the bell... you may wish to switch off your screen until then.
- That is the end of today's task. Please wait while the session completes automatically…

[Next page]

Did you notice anything significant during this session? If so, please provide a description.

When you are finished, click the button below to finish for today.

**Wording for control group instructions**

Having completed your first visit to the laboratory, you will now begin the daily task.

Each day, you will begin by answering several questions. The questions encourage reflection on your emotional responses since your previous log-in. In this way, you can use these questions to document any experiences that have affected you personally. You should feel free to write openly, since your responses will be viewable only by the researchers.

Please try to do the task at the same time each day so that it becomes a habit. Ideally, this should be in the evening, after dinner, so you are not distracted by hunger, long enough after dinner so that you do not feel full but not so long after that it is nearly bedtime. Between 8pm and 10pm would be ideal, depending on your schedule. Those on shift-work may need to adjust these times accordingly.

You will complete this daily task 6 days per week, for eight weeks, followed by a second (and final) laboratory visit. At the 4-week midpoint, the lead researcher (Steven Davey) will be in touch (by Skype/Facetime, or email if you would prefer) to see how you are getting on and to answer any questions. If you experience difficulties in doing the task and feel you need further support before the 4-week midpoint, please contact Steven Davey (davst397@student.otago.ac.nz).

To support you throughout the project, the researcher will check-in regularly with this website to ensure the daily task is running smoothly and to encourage a sense of connection for the 8-week duration. Your efforts will not go unnoticed!

In addition, please note that being allocated to the control group does not mean you will miss out on the meditation task. At the end of the 8 weeks - if you wish - you will be provided with the same access to the meditation program and your activity will be monitored and supported in the same way as the experimental groups. Your access to this will simply be delayed. Regardless of the fact that you will not be doing the meditation task during the study period itself, the value of the compensatory vouchers will be the same for all participants.

Please click the button below to continue.

[The same self-reflection questions are then presented as for the experimental groups; see above]
