# Supplementary material for: Where is an emotion? Using targeted visceroception as a method of improving emotion regulation in healthy participants to inform suicide prevention initiatives: a randomised controlled trial
Source: Trials. 2020 Jul 14;21:642. doi: 10.1186/s13063-020-04479-9 (PMC7362633; doi:10.1186/s13063-020-04479-9)
Supplement: Supplementary file 2 — Additional file 2. Stimuli selected for the tasks [file 13063_2020_4479_MOESM2_ESM.docx]

Appendix 2 – stimuli selected for the tasks

**Spatial cueing task**

*IAPS image identifiers*

Practice block

2102, 2390, 2393, 5725, 5726, 7036, 7061, 7080

Main block

1616, 1675, 2026, 2352.2, 2377, 2381, 2396, 2397, 2411, 2446, 2495, 2512, 2514, 2635, 2703, 2717, 2811, 3000, 3001, 3005.1, 3010, 3015, 3016, 3030, 3051, 3053, 3059, 3060, 3062, 3063, 3064, 3068, 3069, 3071, 3080, 3100, 3101, 3102, 3103, 3110, 3120, 3130, 3131, 3140, 3150, 3168, 3170, 3180, 3191, 3195, 3225, 3266, 3500, 3530, 3550.1, 5395, 5510, 5533, 6022, 6212, 6230, 6243, 6260, 6312, 6313, 6350, 6360, 6415, 6520, 6540, 6560, 6563, 6570, 6831, 6838, 7001, 7002, 7016, 7017, 7018, 7019, 7021, 7026, 7034, 7035, 7040, 7045, 7050, 7056, 7057, 7059, 7062, 7130, 7160, 7170, 7179, 7182, 7185, 7235, 7237, 7242, 7247, 7248, 7249, 7255, 7380, 7547, 7550, 7830, 8312, 9040, 9075, 9163, 9183, 9185, 9187, 9250, 9252, 9253, 9254, 9322, 9325, 9326, 9405, 9410, 9412, 9413, 9414, 9420, 9428, 9429, 9433, 9560, 9570, 9571, 9635.1, 9800, 9810, 9901, 9902, 9903, 9904, 9910, 9911, 9920, 9921, 9940.

*NAPS image identifiers*

Main block

Animals_008_v, Animals_016_h, Animals_039_h, Animals_056_h, Animals_063_h, Animals_074_h, Animals_077_h, Faces_009_h, Faces_010_h, Faces_016_h, Faces_018_h, Faces_031_v, Faces_143_v, Faces_145_v, Faces_147_v, Faces_149_v, Faces_153_v, Faces_159_h, Faces_170_h, Faces_172_h, Faces_174_h, Faces_283_h, Faces_284_h, Faces_293_h, Faces_362_v, Faces_364_v, Faces_365_v, Faces_366_h, Faces_367_h, Faces_371_v, Landscapes_022_h, Objects_001_h, Objects_003_h, Objects_149_h, Objects_283_h, People_001_h, People_003_h, People_016_h, People_020_h, People_031_v, People_038_h, People_127_h, People_128_h, People_198_h, People_200_h, People_201_v, People_202_h, People_204_v, People_205_v, People_208_h, People_211_v, People_216_h, People_218_v, People_220_h, People_221_h, People_225_h, People_226_h, People_227_h, People_237_h, People_238_h, People_240_h, People_241_h, People_242_v, People_243_h, People_246_h.

**Stop/signal task**

*IAPS image identifiers*

Practice block

5635, 7025, 7033, 7090, 7140, 7150, 7233, 7509.

Main block

1710, 1811, 1999, 2002, 2038, 2045, 2071, 2091, 2102, 2150, 2158, 2209, 2210, 2214, 2216, 2300, 2340, 2345.1, 2357, 2385, 2390, 2393, 2398, 2484, 2595, 2745.1, 2800, 2890, 3061, 3160, 3215, 3220, 3230, 4599, 4626, 4641, 5040, 5270, 5460, 5470, 5480, 5600, 5623, 5700, 5740, 5825, 5829, 5830, 5833, 5910, 6150, 6570.2, 6825, 7000, 7003, 7004, 7006, 7009, 7010, 7012, 7014, 7020, 7037, 7041, 7052, 7053, 7055, 7061, 7080, 7100, 7175, 7187, 7207, 7492, 7500, 7502, 7512, 7705, 8120, 8170, 8190, 8210, 8420, 8470, 8496, 8499, 8502, 8540, 9140, 9181, 9301, 9302, 9332, 9520, 9900.

*NAPS image identifiers*

Main block

Animals_013_h, Animals_024_h, Animals_025_h, Animals_033_h, Animals_048_h, Animals_054_h, Animals_062_h, Animals_068_h, Animals_071_h, Animals_078_h, Faces_003_h, Faces_019_h, Faces_028_h, Faces_152_h, Faces_368_h, Objects_132_h, Objects_139_h, Objects_285_h, People_004_h, People_009_h, People_013_v, People_021_h, People_075_v, People_235_h, People_239_h.
