# Supplementary material for: Where is an emotion? Using targeted visceroception as a method of improving emotion regulation in healthy participants to inform suicide prevention initiatives: a randomised controlled trial
Source: Trials. 2020 Jul 14;21:642. doi: 10.1186/s13063-020-04479-9 (PMC7362633; doi:10.1186/s13063-020-04479-9)
Supplement: Supplementary file 3 — Additional file 3. Consent form [file 13063_2020_4479_MOESM3_ESM.docx]

Appendix 3 – Consent Form

**[Reference Number: 18/168]**

**[Date: 24/10/2018]**

**Where is an emotion?**

CONSENT FORM FOR PARTICIPANTS

I have read the Information Sheet concerning this project and understand what it is about. All my questions have been answered to my satisfaction. I understand that I am free to request further information at any stage.

I know that:-

1. My participation in the project is entirely voluntary.

2. I am free to withdraw from the project before its completion (July/August 2019), and to withdraw all data I provide (before the end of September 2019 by which time analysis will have been completed).

3. Personal identifying information (from audio recordings) will be destroyed at the conclusion of the project but any other raw data on which the results of the project depend will be retained in secure storage for at least five years.

4. For those who choose to participate in a semi-structured interview, this project involves an open-questioning technique. The general line of questioning includes your experiences of emotion before and during the project and any changes you may have identified. The precise nature of some of the questions which may be asked have not been determined in advance, but will depend on the way in which the interview develops and that in the event the line of questioning develops in such a way that I feel hesitant or uncomfortable I may decline to answer any particular question(s) and/or may withdraw from the project without any disadvantage of any kind.

5. Possible discomforts, risks or inconveniences include: the use of images designed to elicit strong emotional responses, self-report questionnaires that ask about emotional state, water intake leading to a feeling of fullness, extended periods of time viewing a computer screen, and the daily 20-minutes of effort when paying focused attention.

6. Participants will each receive a $150 voucher as compensation for their effort in completing the project. In addition, 15 of these participants will take part in a one-hour interview, and will receive an additional $20 voucher as compensation for completing this.

7. The results of the project may be published and will be available in the University of Otago Library (Dunedin, New Zealand) but every attempt will be made to preserve my anonymity.

I agree to take part in this project.
